# Near-Infrared Tunable Laser Absorption Spectroscopic Acetylene Sensor System Using a Novel Three Mirror-Based, Dense Pattern Gas Cell

**DOI:** 10.3390/s20051266

**Published:** 2020-02-26

**Authors:** Guoqiang Zhong, Zhuo Ma, Junbo Wang, Chuantao Zheng, Yu Zhang, Yiding Wang, Frank K. Tittel

**Affiliations:** 1 State Key Laboratory of Integrated Optoelectronics, College of Electronic Science and Engineering, Jilin University, 2699 Qianjin Street, Changchun 130012, China; zhonggq17@mails.jlu.edu.cn (G.Z.); mazhuo19@mails.jlu.edu.cn (Z.M.); wangjb18@mails.jlu.edu.cn (J.W.); yuzhang@jlu.edu.cn (Y.Z.); ydwang@jlu.edu.cn (Y.W.); 2 Department of Electrical and Computer Engineering, Rice University, 6100 Main Street, Houston, TX 77005, USA; fkt@rice.edu

**Keywords:** multipass gas cell, acetylene detection, wavelength modulation spectroscopy, laser absorption spectroscopy

## Abstract

By contrast with the widely reported traditional two mirror-based Herriott cell, a three mirror-based dense pattern gas cell was proposed, of which the modeling and design were proven to be effective through a comparison between the simulated spot pattern and effective path length and those of the experimental results. A mechanical structure was designed to adjust the position/angle of the three mirrors for aligning the optical path. The experimentally measured reflection number was 60, resulting in an optical path length of ~11 m, which agrees well with the theoretical value of 10.95 m. Combined with a near-infrared laser with a center wavenumber located at an acetylene (C_2_H_2_) absorption line of 6521.2 cm^−1^, a C_2_H_2_ sensor system was established to verify the feasibility of the three mirror-based gas cell. Assisted by a data acquisition (DAQ) card, a LabVIEW platform was developed to generate the drive signal of the laser and acquire the second harmonic (2*f*) signal from the output of the detector. Through Allan variance analysis, the limit of detection (LoD) of the sensor system is 4.36 ppm at an average time of 0.5 s; as the average time exceeds 10 s, the LoD is <1 ppm. The proposed model and design of the three mirror-based gas cell can be used to realize similar gas cells with different absorption path lengths for gas detection based on infrared absorption spectroscopy.

## 1. Introduction

There are various kinds of gas species in the atmospheric environment of human activity. Some harmful gases are toxic and flammable, e.g. acetylene (C_2_H_2_) and methane (CH_4_) [1,2,3], and they can even cause explosions when the concentration level exceeds the explosion limit. In order to avoid safety accidents, gas detection is particularly important. The available gas detection techniques mainly include infrared absorption spectroscopy, catalytic combustion, and electrochemical approaches [4,5,6,7]. Infrared absorption spectroscopy has the characteristics of high sensitivity, fast response and high stability. Tunable diode laser absorption spectroscopy (TDLAS), an absorption spectroscopy technique, has fast response, high precision, good single-mode characteristics and strong adaptability [8,9,10,11,12]. Moreover, the combination of wavelength modulation spectroscopy (WMS) can improve the detection sensitivity of TDLAS, which is widely used in infrared gas sensor systems.

Based on the Lambert–Beer law, infrared absorption spectroscopy reveals the relationship between light intensity, gas concentration and optical path length. Increasing optical path length can effectively improve the detection sensitivity, where gas cell is a key module in such a sensor. Two classical gas cells are usually used to increase the optical path length: a White cell and a Herriot cell [13,14,15,16]. A White cell uses three spherical mirrors to form a conjugate system, while a Herriot cell uses two parallel spherical mirrors. The distance between the mirrors of a White cell should be the same as the radius of curvature to form a conjugate system, so that the spots are symmetrically distributed up and down. Therefore, the path length is determined by the number of reflections, which is limited by the size of the mirror. For obtaining a long optical path length, it is necessary to select a mirror with a large focal length or a large size, and thus the volume of the gas cell cannot be reduced. Under the condition of paraxial incidence, according to the ABCD optical matrix [17], an elliptically distributed spot pattern can be obtained on the mirror. Furthermore, in recent years, many new types of gas cell were reported. In 2014, Mu et al. proposed a new type of White cell with symmetrical upper and lower spot pattern [18]. The spot distribution is simple and easy to adjust; however, it is not easy to fabricate a rectangular mirror, the number of reflections is limited, and with a long optical path, the cell volume is too large. In 2018, Dong et al. demonstrated a double-ring Herriot gas cell with two optical path lengths, which can be used to measure two gas species or for the detection of a single gas with two measurement ranges [19]. The disadvantage of this cell is that the mirror utilization rate is low and the cell volume is too large. In 2019, Zheng et al. proposed an acetylene sensor based on off-axis integrated-cavity output spectroscopy (OA-ICOS) [1]. The optical path length is greatly affected by the mirror reflectivity. A large optical path length requires an expensive, highly reflective mirror. 

In this work, a new type of gas cell structure is proposed and an acetylene sensor system is reported based on the proposed gas cell. By contrast with the traditional two mirror-based gas cell, the position and angle of the three mirrors are flexible because of the use of three spherical mirrors. Compared with previously reported double-ring Herriott gas cell [19], the three mirror-based gas cell has a large spot number and dense spot distribution. Therefore, the three mirror-based gas cell can realize larger optical path length with small mirror size and cell volume. Moreover, the maximum reflection number is achieved when the size of the spot-ring equals the maximum radius of the mirror. The differences of this cell structure are described as below. Firstly, the incident light does not need to be paraxial, which shortens the distance between the mirrors and thus reduces the cell volume and improves the cell stability. Secondly, the reflection of the traditional two mirror-based cell only exists in one dimension. However, another dimension is added in the three mirror-based cell, which leads to a triangular optical reflection and a long optical path with the same cell volume. Finally, with appropriate optimization, the light spot can be distributed on the whole mirror surface, which improves the utilization rate of the mirror. Under the same targeted optical path length, the mirror size and cell volume can be reduced. In other words, under the same cell volume, a long optical path can be achieved for enhancing the sensor sensitivity.

## 2. Three Mirror-Based Gas Cell

### 2.1. Gas Cell Structure

**Figure 1:**
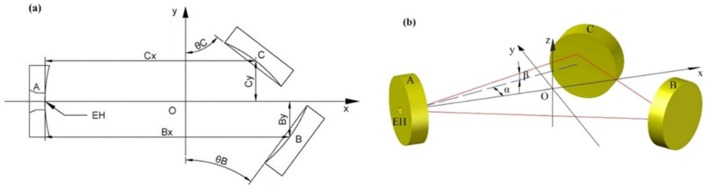
(**a**) The structural parameters in the XOY plane and (**b**) the three-dimensional CAD model of the three-mirror-based gas cell.

The structure of the three mirror-based gas cell is shown in Figure 1a. The spherical center of mirror A is set as the coordinate origin, the direction perpendicular to the mirror A is the *x*-axis, the direction parallel to the mirror A is the *y*-axis, and the vertical direction is set to the *z*-axis. Figure 1b shows a three-dimensional model of the gas cell designed by Auto computer aided design (CAD) software. There is an entrance hole (EH) on mirror A. The direction angle and pitch angle of the entrance light are defined as *α* and *β*, respectively. The central distance projected to the x axis between mirror A and mirror C is *C*_x_ and that to the y axis is *C*_y_. Similarly, the central distance projected to the x axis between mirror A and mirror B is *B*_x_ and that to the y axis is *B*_y_. The angle between mirror C and the y axis is *θ*_C_, and that between mirror B and the y axis is *θ*_B_. The incident light propagates through the EH on mirror A to mirror C and reflects to mirror B. After a certain number of reflections, the light reflects from mirror C and stops reflection after propagating through the EH of mirror A.

In the modelling and simulation of the gas cell, the three mirrors have ideal sphere shape, the center points of the mirrors are located in the same plane which is perpendicular to the horizontal plane, and the effect of the thickness of the mirror is not considered. The incident ray is assumed to be a straight line, regardless of the effects of the angle of the exit ray and the thickness of the mirror on the ray emission. Also, it is assumed that the spot diameter of the laser beam is 1 mm. Light obeys the law of reflection, the reflection angle is equal to the incident angle, and the incident ray, normal line and reflection ray are in the same plane.

### 2.2. Simulation

When the center of the sphere is (*x*_A_, *y*_A_, *z*_A_) and the focal length of the mirror is *f*, the equation of the mirror A can be described as:

(1) $${\left(x-x_{A}\right)}^{2}+{\left(y-y_{A}\right)}^{2}+{\left(z-z_{A}\right)}^{2}={\left(2f\right)}^{2}$$

The unit normal vector (*m*, *n*, *k*) describing the equation of the line can be obtained from the angle, combining the coordinates of the incident point (*x*_0_, *y*_0_, *z*_0_), the equation of the ray can be described as:

(2) $$\frac{x-x_{0}}{m}=\frac{y-y_{0}}{n}=\frac{z-z_{0}}{k}$$

By combining Equations (1) and (2), we can obtain the intersection of the ray and the sphere. When the obtained intersection point is located within the mirror, the reflection is valid. Then the angle of the reflection ray is determined by the law of reflection, and the equation of the reflected ray can be determined. The reflection stops until the desired point of intersection is perforated at the exit of mirror A. In addition, there may be no solution or the intersection point is not on the mirror, and this case will be unconsidered. Also, we added the maximum number of reflections as a loop-stop condition of the program.

**Figure 2:**
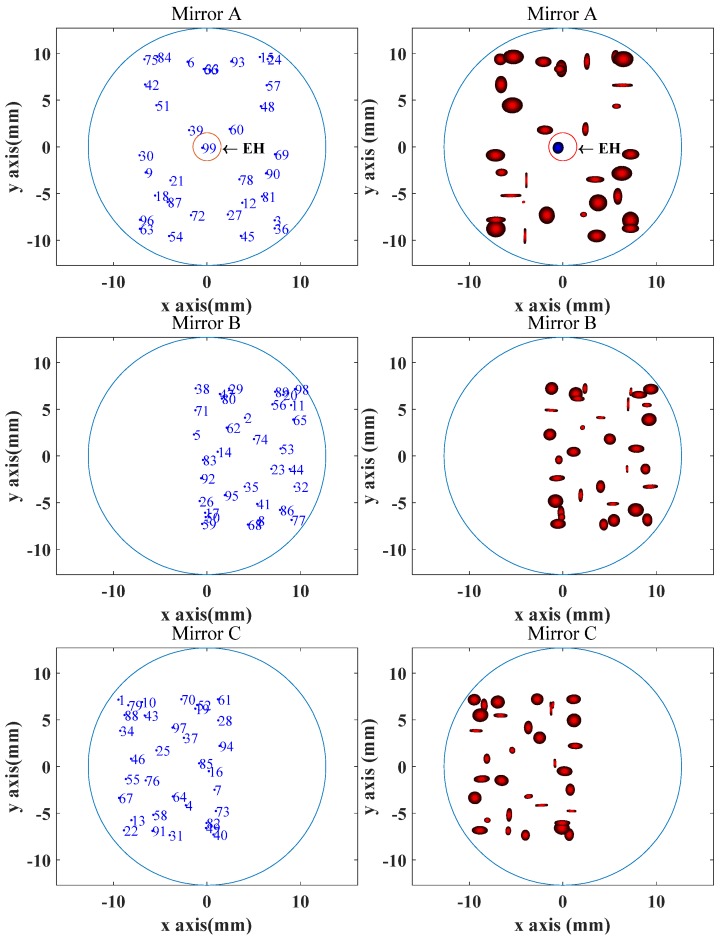
The simulated reflection number and spot distribution on mirror A, B and C.

Considering the actual size, the spot cannot be near the edge of the mirror or exit partially from the EH during reflection. With an incident laser beam diameter of 1 mm, a simulation program was designed to check whether the position and shape of the reflected spot meet the requirements or not. As shown in Figure 2, the number of reflections and the spot shape on each mirror are obtained under the used simulation parameters in Table 1. When the elevation angle of the incident light increases, the spot will disperse up and down; when the direction angle of the incident light increases, the spot will disperse left and right. Under the same parameters of the mirror, if the incident angle increases, the light continues reflecting among the mirrors which increases the reflection number. However, when the reflected light reaches the boundary of the mirror, the angle cannot be increased any more. By contrast with the incident circular spot, after reflection between the anti-spherical mirrors, the light beam converges and diverges, making the shape of the spot change during reflection.

Since the number of adjustable parameters of the three mirror-based gas cell is more than that of the traditional two mirror-based Herriott cell, it is more meaningful to study the influence of the distance and angle between mirrors on the optical path length and spot distribution. In the selection of mirrors, concave mirrors with convergent function should be used. We tried using an off-axis parabolic mirror to replace one of the mirrors, but it is hard to obtain a long optical length.

### 2.3. Mechanical Stability Analysis of the Gas Cell

**Figure 3:**
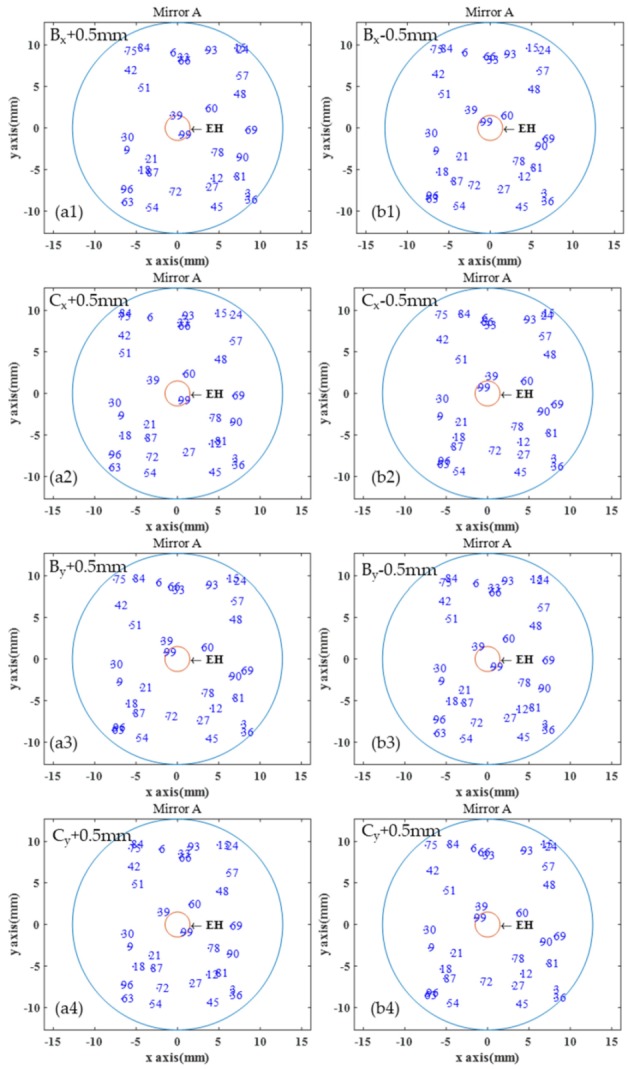
The simulated reflection number and spot distribution on mirror A with (**a1**) *Bx* + 0.5 mm, (**b1**) *Bx* − 0.5 mm, (**a2**) *Cx* + 0.5 mm, (**b2**) *Cx* − 0.5 mm, (**a3**) *By* + 0.5 mm, (**b3**) *By* − 0.5 mm, (**a4**) *Cy* + 0.5 mm, (**b4**) *Cy* − 0.5 mm.

When the optical path is affected by mechanical vibration, the stability of the optical path is particularly important. Assume that there is an offset added to the simulation parameters in Table 1 to observe the change in the mode pattern. Figure 3 shows the spot distribution on Mirror A with *B*_x_, *C*_x_, *B*_y_, and *C*_y_ increased or decreased by 0.5 mm. The mechanical vibration does not affect only one parameter. The variation of each parameter depends on the vibration intensity. From the simulation result, when there is a slight change in a single parameter, the spot position on Mirror A will not be significantly shifted, and the reflection number and the optical path will not be affected.

### 2.4. Comparison with the Two Mirror-Based Herriott Cell

**Figure 4:**
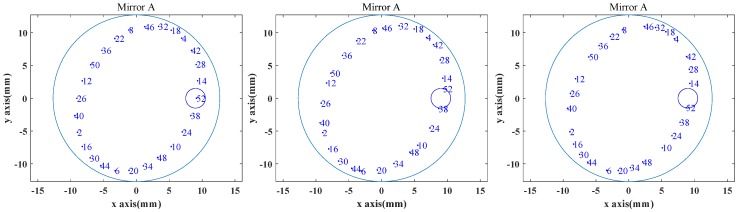
The spot distribution of a two mirror-based Herriott cell on Mirror A with (**a**) *D*, (**b**) *D* + 0.5 mm and (**c**) *D* − 0.5 mm.

**Figure 5:**
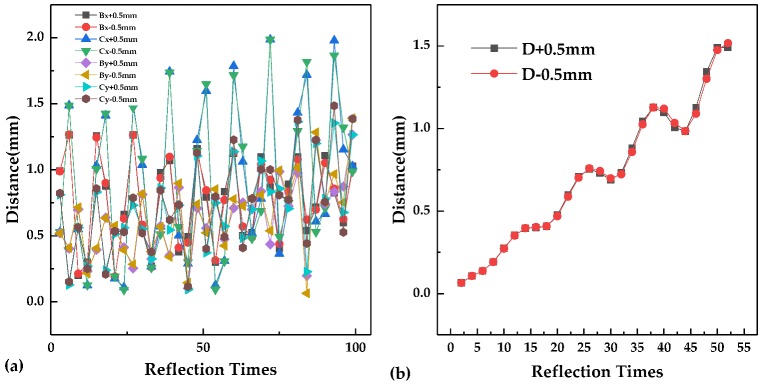
(**a**) Relation between the shift distance of the exit spot on mirror A and the reflection times before and after changes added to *B*_x_, *B*_y_, *C*_x_, *C*_y_ for the three mirror-based gas cell. (**b**) Relation between the shift distance of the exit spot on mirror A and the reflection times before and after changes added to *D* for the two mirror-based gas cell.

A MATLAB program was used to simulate a traditional Herriott cell with the parameters in Table 2, the model of which can be seen in Reference 19. *D* is the distance between the two Herriott mirrors, *f* is the focus of the mirrors, the entrance position is (*x*, *y*, *z*) on mirror A, and the entrance angles are *α* and *β*. The spot distribution obtained is shown in Figure 4. Figure 4a shows the spot distribution of a two-mirror-based Herriott cell on Mirror A under the designed parameters in Table 2. The exit spot #52 is located at the center of the exit hole, and the optical path is determined to be 12.9 m. Figure 4b is the spot distribution with *D* increased by 0.5 mm, and Figure 4c shows the spot distribution with *D* reduced by 0.5 mm. Figure 5 shows the relation between the shift distance of the exit spot on mirror A and the reflection times before and after changes added to *B*_x_, *B*_y_, *C*_x_, *C*_y_ or D for the three mirror-based gas cell and the two mirror-based gas cell. For the two mirror-based Herriott cell, the spot shift distance increases significantly with the number of reflections. The offset distance obtained by the proposed scheme is less affected by the reflection number and is more suitable for achieving a long optical path and a good stability.

## 3. Gas Cell Development and Measurement

### 3.1. Mechanical Structure Design

**Figure 6:**
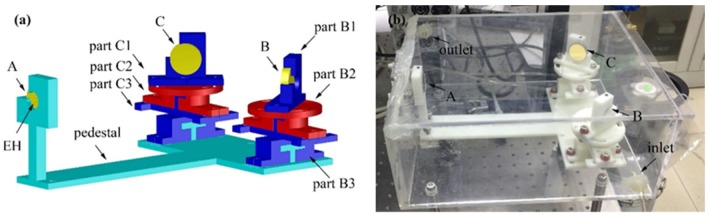
(**a**) A 3D model of the mechanical adjusting structure. (**b**) A photo of the developed gas cell for gas detection with a gas inlet and an outlet.

The parameters of the gas cell need a high accuracy to adjust, and therefore it is necessary to design a mechanical structure to assist in adjusting the angles and positions of the three mirrors. According to the simulation parameters of the chamber, a mechanical structure was required, by which the position of the mirror can be changed (±10 mm) and the angle can be rotated by 90°. A 3D model of the mechanical adjust structure is shown in Figure 6a. With a reference to the position of mirror A, the blue elements (part B3 and C3) can adjust the parameters of *B*_y_ and *C*_y_, the red elements (part B2 and C2) can adjust the parameters of *B*_x_ and *C*_x_, and the blue mirror frame (part B1 and C1) can adjust the parameters of *θ*_B_ and *θ*_C_ by 90°. Each part of the adjusting structure can change only one parameter without affecting other parameters. The mechanical structure was fabricated and assembled by using a 3D printing technique, and had a size of 247 × 160 × 90 mm^3^. The mirror with a reflectance of ~98.28% near the wavelength of 1.53 μm was fixed on the mirror frame, and the position was adjusted by observing the mode pattern of the red trace laser. A final structure was obtained with the target spot distribution observed. Furthermore it should be noted that (1) reducing the cell volume can directly improve the response speed of the sensor; (2) a small-volume cell can reduce the total amount of the target gas; (3) a sensor can be made portable with the small cell; (4) The resistance of the size-reduced sensor to mechanical vibration can be improved.

In the cell model, the three mirrors were placed vertically with the same height. But in actual fact, it became a problem to ensure the height of each adjusting elements. The error between the heights of the adjusting elements has less effect on the spot distribution than the parameters between mirrors.

Then the mechanical structure with the three mirrors was fixed in an acrylic sealed box as a gas chamber for gas measurement. There is a window on the front surface of the chamber (WG5150, Thorlabs, Newton, NJ, USA), which allows the laser beam to enter the chamber for reflection. The inlet and outlet air holes are located at both sides of the air chamber. A photo of the developed gas cell is shown in Figure 6b whose dimension size is 287 × 241 × 115 mm^3^.

### 3.2. Gas Cell Measurement

**Figure 7:**
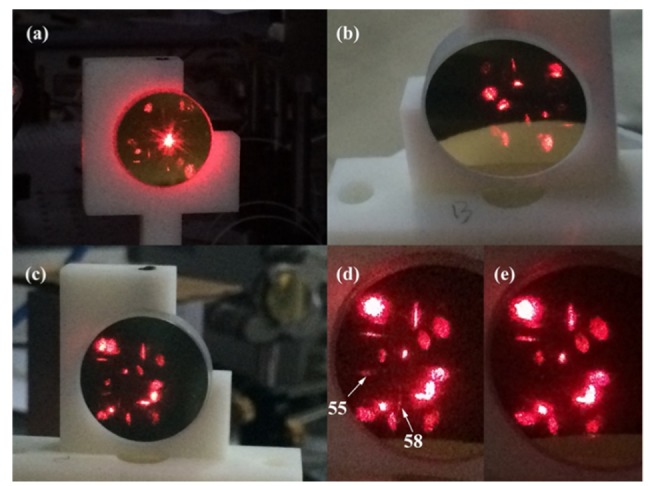
The measured spot shape and distribution on (**a**) mirror A, (**b**) mirror B, and (**c**) mirror C for the optimized three mirror-based gas cell. (**d**) The 55th and 58th spots on mirror C. (**e**) Spot distribution on mirror C after blocking the 54th spot.

The parameters of the gas cell were tuned until the desired spot pattern was obtained. The image of the observed spot is shown for mirror A, B and C in Figure 7. The pattern obtained shows good agreement with the simulated results shown in Figure 2, which proves the accuracy of the gas cell model and theory.

The number of reflections determines the optical length, but the number of visible spots does not determine the optical length. However, the spot distribution can be used for the adjustment of the optical path, and the optical length needs to be measured accurately through the optical length calibration experiment. Therefore, as long as the number of the visible spots reaches the expected value, the optical length can meet the design requirement. In the system, the red laser was used to adjust the optical path, and the spot distribution can be observed directly. By blocking some spot and observing the residual spot shape, such as the 21st spot and the 18th spot on mirror A, which are in a thin strip perpendicular to each other, we can judge whether the optical length meets the design requirement. If the number of reflections increases, the intensity of the beam gets weak to a certain extent, and a high-power laser is preferred. If the number of spots is too large, image analysis can be considered to identify the number of spots by boundary extraction and other algorithms.

Then we determined the effective absorption path length of the gas cell under this configuration. As shown in Figure 7d,e, when the 54th spot on mirror A is blocked, the 55th and 58th spot on mirror C disappear, while the other spots on mirror C remain unchanged, indicating that the number of reflections is ~60. This results in an optical path length of 10.95 m with 60 reflections.

In the simulation, the reflection number is 99, and the number obtained in the experiment is much less than that in the simulation. As can be seen from Figure 2, the 60th spot on mirror A was very close to the EH. Due to the accumulation of errors in various parameters, the 60th spot was ejected from the incoming perforation, causing the reflection to stop. Efforts were made to increase the reflection number but it is difficult to do so since there is no specific formula for the relation between the gas cell parameters and the spot distribution.

By blocking the spot, the reflection number of the spot can be observed. By blocking the 54th spot on mirror A, all spots after spot 54 should disappear. However, only two spots disappeared on mirror C, 55 and 58, and the total number of spots on each mirror was the same; also, two spots disappeared on mirror A, namely 57 and 60. Combined with simulation results, spot 60 was very close to the perforation, so it can be inferred that spot 60 was emitted from the EH. The spot distribution indicates that all the light is emitted after 60 reflections and no light continues to reflect in the air chamber.

**Figure 8:**
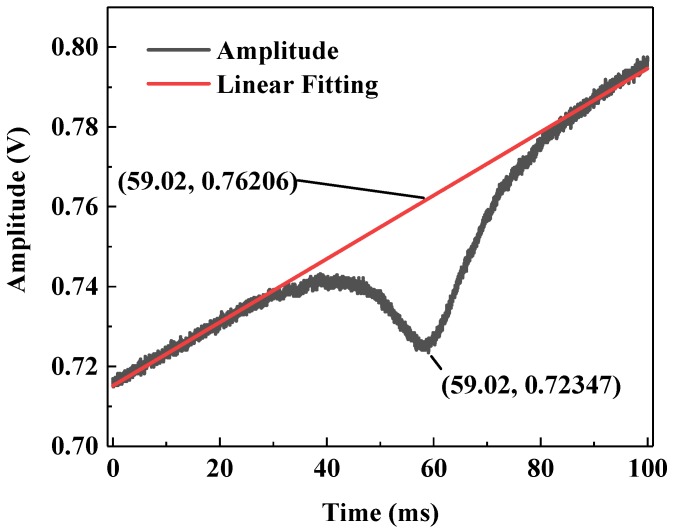
Measured C_2_H_2_ absorption signal (black curve) with the reported three mirror-based gas cell at a concentration level of 2000 ppmv. The red curve shows the background fitting signal.

An experiment was performed to verify the optical path. The optical path length of the chamber was experimentally measured using a C_2_H_2_ sample with a concentration level of 2000 ppm. The drive signal of the distributed feedback (DFB) laser is a 5 Hz sawtooth signal. When there is no acetylene gas in the chamber, the output signal from the detector should be a 5 Hz triangular wave. When the 2000 ppm acetylene is injected, the optical power received by the detector decreases. The measured output signal from the detector is shown in Figure 8. The maximum attenuation voltage was 0.723 V (*V*_1_), and the original signal voltage without acetylene absorption was 0.762 V (*V*_2_), which was derived from the linear fitting curve. The bias voltage of the detector was 0.688 V (*V*_0_) without laser irradiation. Therefore, according to Equation (3), it is written as:

(3) $$\alpha =ln\left(\left(V_{2}-V_{0}\right)/\left(V_{1}-V_{0}\right)\right)$$

and the absorbance (a) is calculated to be 0.736. Compared to the simulation result on the SpectraPlot website [20], the obtained optical path length is therefore determined to be 11.0 m, which is in good agreement with the theoretical result of 10.95 m.

## 4. Performance of the C_2_H_2_ Sensor

### 4.1. Sensor Configuration

**Figure 9:**
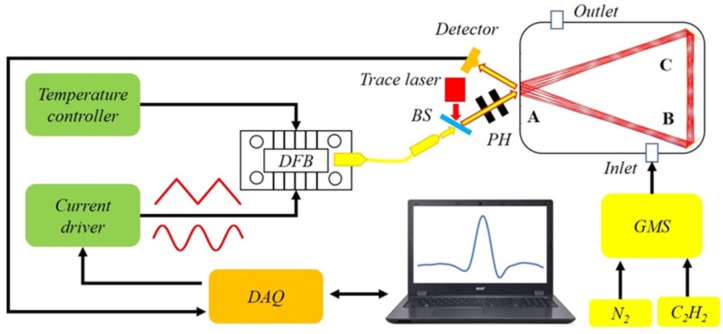
Schematic of the near-infrared C_2_H_2_ sensor system using the three mirror-based gas cell, including an electrical system, an optical system, as well as a gas sampling system.

As shown in Figure 9, the structure of the sensor system is similar to the system previously reported by our group [19]. The three mirror-based gas cell was used instead of the traditional Herriott cell [19], and only one detector was adopted for single-gas measurements. With a gas mixing system (GMS, Series 4000, Environics, Tolland, CT, USA), 2000 ppm C_2_H_2_ and pure N_2_ were used to produce gas samples with different concentration levels for experiments. The specific parameters of the system are summarized and shown in Table 3.

With a data acquisition (DAQ) card, a LabVIEW platform was developed to control the current and temperature of the laser. The optical path was aligned with a visible trace laser. The output laser beam was overlapped with the red laser beam through a beam splitter (BS) and two pinholes (PH). The combined beam enters the multi-pass cell and reaches the detector after multiple reflections. The output signal from the detector was delivered to the LabVIEW platform for post data processing and analysis.

### 4.2. Calibration and Data Fitting

**Figure 10:**
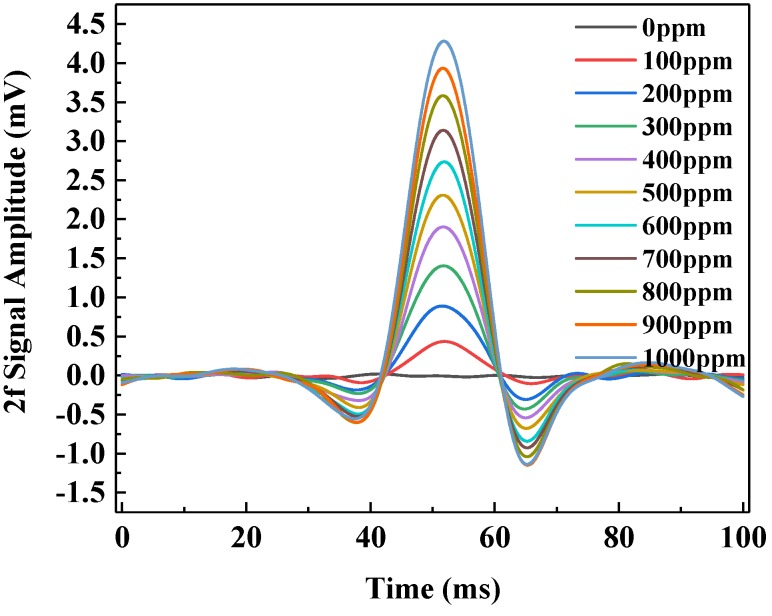
The observed 2*f* signals in the C_2_H_2_ concentration range of 0–1000 ppm.

The output signal of the detector is transmitted to the laptop for 2*f* signal extraction based on LabVIEW. The C_2_H_2_ gas samples in the concentration range of 0–1000 ppmv with a step of 100 ppmv were prepared using the gas mixing system. Within the concentration range of 0–1000 ppm, the measured 2*f* signals for the gas samples with different concentration levels are shown in Figure 10.

**Figure 11:**
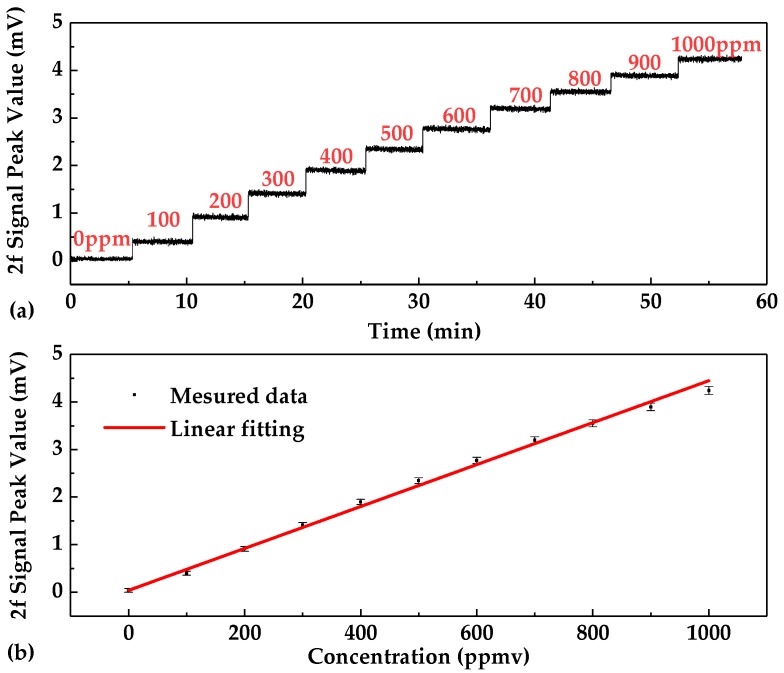
(**a**) Measured 2*f* signal amplitude versus calibration time t for different C_2_H_2_ concentration levels ranging from 0 to 1000 ppmv. (**b**) Experimental data dots and fitting curve of C_2_H_2_ concentration versus the 2*f* signal amplitude.

Because the peak value of the 2*f* signal is linear with the concentration [21], the 2*f* signal amplitude was used to calibrate the sensor. As shown in Figure 11a, each gas sample was measured for ~5 min, and ~800 data points were obtained and then the mean value of the 2*f* signal amplitude was calculated and plotted as a function of C_2_H_2_ concentration. The linear relationship between the concentration (*C*) and the second harmonic peak (2*f* signal amplitude) is shown in Figure 11b. A fitting linear equation is observed and shown by Equation (4) (R-square value: 99.88%), given by:

(4) 2f_PeakValue(mV)=0.00437×C(ppm)+0.03995

The fitting curve indicates a good linear relationship (R-square value: 99.88%) between the 2*f* amplitude and C_2_H_2_ concentration. The different errors for different concentration levels may result from the gas-mixing system for preparing different gas samples.

### 4.3. Sensor Stability

**Figure 12:**
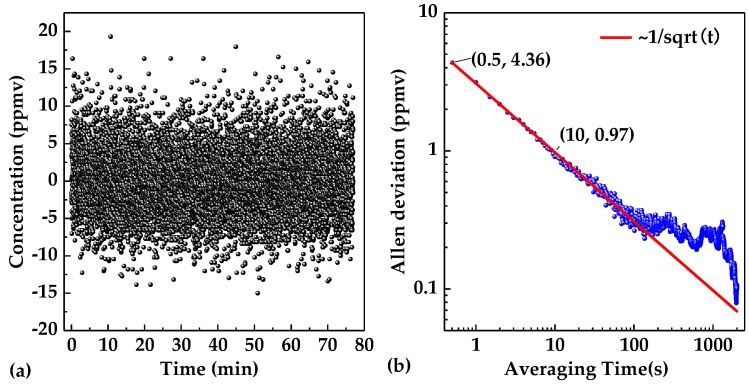
(**a**) C_2_H_2_ concentration measurements of the sample with zero concentration for a time period of >1 hour. (**b**) Allan deviation plot of the C_2_H_2_ sensor with a sampling interval of 0.5 s based on the data shown in Figure 12a.

Sensor stability can be analyzed by carrying out an Allan deviation analysis on the sensor system. N_2_ was injected into the gas chamber for 2 h, and meanwhile, the 2*f* peak values were acquired and recorded via the LabVIEW platform. The 2*f* peak values were then converted into concentration levels according to Equation (4), as shown in Figure 12a. Under a C_2_H_2_ concentration level of 0 ppmv, the variation range of the acetylene sensor reading was from −15 ppmv to 20 ppmv. The noise of the sensor system leads to a fluctuation range of the measurement result, which can be reduced by averaging. Therefore Allan deviation can be used to analyze the limit of detection (LoD) under different averaging time. The LoD for different averaging time (*τ*) can be calculated by Allan deviation, as shown in Figure 12b. When the averaging time was 0.5 s, the LoD of the system was ~4.36 ppmv; when the averaging time exceeded 10 s, the LoD could be lower than 1 ppm. The Allan deviation decreased obeying the rule of ~1/*√τ* with *τ* ≤ 100 s. This indicated that the dominated noise in the system is White–Gaussian noise that can be removed by simply using the data averaging method.

## 5. Conclusions

A novel three mirror-based dense-pattern gas cell was proposed for gas detection based on infrared absorption spectroscopy. The gas cell had a smaller volume and a higher utilization rate due to the use of three mirrors than the traditional Herriott gas cell based on two mirrors. In order to accurately tune the positions and angles of the three mirrors, a novel mechanical structure was fabricated. A C_2_H_2_ sensor system was presented using this gas cell by targeting the line located at 6521.2 cm^−1^. Using the 2*f*-based WMS technique, C_2_H_2_ measurements were carried out to validate the accuracy of the modeling and formulation of the three mirror-based dense pattern gas cell. Future work is still required to find the rule of spot distribution on the three mirrors, which is important for improving the utilization rate of the mirrors and enlarging the optical path length with a small cell volume and a high reflection of the mirror.

## Figures and Tables

**Table 1:** The parameters used in the simulation in Figure 2.

Parameters	B_x_	B_y_	C_x_	C_y_	θ_B_	θ_C_	α	β
Value	201mm	63 mm	201 mm	63 mm	53°	53°	19.5°	2°

**Table 2:** The parameters used in the simulation of Herriott.

Parameters	D	f	x	y	z	α	β
Value	248 mm	100 mm	−199.7 mm	9 mm	0 mm	−2.1°	2.5°

**Table 3:** The parameters and models of the key modules used in the sensor system.

Module	Laser	Laser Current Driver and Temperature Controller		
	Wavenumber range	Model	Current range	Temperature
Value/Type	6520.4−6522.0 cm^−1^	LDTC0520, Wavelength Electronics, USA	38–96 mA	23.8 °C
**Module**	**Detector**	**Data acquisition card**		
	**Model**	**Model**	**Sampling rate**	
Value/Type	PDA10, Thorlabs, USA	USB-6211, National Instrument, USA	100 kHz	
